# Interannual and Seasonal Dynamics of Volatile Organic Compound Fluxes From the Boreal Forest Floor

**DOI:** 10.3389/fpls.2019.00191

**Published:** 2019-02-22

**Authors:** Mari Mäki, Juho Aalto, Heidi Hellén, Mari Pihlatie, Jaana Bäck

**Affiliations:** ^1^Institute for Atmospheric and Earth System Research/Forest Sciences, Helsinki, Finland; ^2^Department of Forest Sciences, Faculty of Agriculture and Forestry, University of Helsinki, Helsinki, Finland; ^3^Department of Physics, Faculty of Science, University of Helsinki, Helsinki, Finland; ^4^Finnish Meteorological Institute, Helsinki, Finland; ^5^Department of Agricultural Sciences, Faculty of Agriculture and Forestry, University of Helsinki, Helsinki, Finland; ^6^Viikki Plant Science Centre, University of Helsinki, Helsinki, Finland

**Keywords:** biogenic volatile organic compound, flux, forest floor, temperature, seasonality, vegetation, decomposition

## Abstract

In the northern hemisphere, boreal forests are a major source of biogenic volatile organic compounds (BVOCs), which drive atmospheric processes and lead to cloud formation and changes in the Earth’s radiation budget. Although forest vegetation is known to be a significant source of BVOCs, the role of soil and the forest floor, and especially interannual variations in fluxes, remains largely unknown due to a lack of long-term measurements. Our aim was to determine the interannual, seasonal and diurnal dynamics of boreal forest floor volatile organic compound (VOC) fluxes and to estimate how much they contribute to ecosystem VOC fluxes. We present here an 8-year data set of forest floor VOC fluxes, measured with three automated chambers connected to the quadrupole proton transfer reaction mass spectrometer (quadrupole PTR-MS). The exceptionally long data set shows that forest floor fluxes were dominated by monoterpenes and methanol, with relatively comparable emission rates between the years. Weekly mean monoterpene fluxes from the forest floor were highest in spring and in autumn (maximum 59 and 86 μg m^-2^ h^-1^, respectively), whereas the oxygenated VOC fluxes such as methanol had highest weekly mean fluxes in spring and summer (maximum 24 and 79 μg m^-2^ h^-1^, respectively). Although the chamber locations differed from each other in emission rates, the inter-annual dynamics were very similar and systematic. Accounting for this chamber location dependent variability, temperature and relative humidity, a mixed effects linear model was able to explain 79–88% of monoterpene, methanol, acetone, and acetaldehyde fluxes from the boreal forest floor. The boreal forest floor was a significant contributor in the forest stand fluxes, but its importance varies between seasons, being most important in autumn. The forest floor emitted 2–93% of monoterpene fluxes in spring and autumn and 1–72% of methanol fluxes in spring and early summer. The forest floor covered only a few percent of the forest stand fluxes in summer.

## Introduction

Global forest ecosystems are the largest existing source of biogenic volatile organic compounds (BVOCs) (Guenther et al., 1995). BVOC emissions are dominated by isoprene and monoterpenes (Lathière et al., 2005; Guenther et al., 2012), and they have a crucial role in the atmosphere as their oxidation products drive secondary organic aerosol (SOA) formation (Kulmala et al., 1998, 2013). SOA formation from volatile organic compounds (VOCs) through oxidation processes and SOA particles can change the Earth’s radiation budget as a cooling feedback mechanism (Virtanen et al., 2010; Kourtchev et al., 2016). The cooling feedback mechanism could become stronger if the temperature were to increase in high latitudes by an estimated 6–8°C by 2100 (IPCC Fifth Assessment Report (AR5), 2014), boosting SOA load and cloud formation by increasing VOC emissions from the boreal biosphere (Kulmala et al., 2014), which is a well-documented VOC source (Rinne et al., 2007). VOCs affect hydroxyl (OH) radical and ozone formation and oxidation processes (Jacob et al., 2005; Hüve et al., 2007). Ozone formation in photochemical reactions requires NOx and reactive VOCs (Crutzen, 1979; Logan, 1985), and for this reason, it is important to quantify seasonal VOC emission fluxes from different sources. These sources should be quantified more accurately, because several studies have shown that measured and modeled ozone deposition fluxes and OH radical reactivities include significant differences (Mogensen et al., 2011; Wolfe et al., 2011; Rannik et al., 2012; Zhou et al., 2017). A gap in atmospheric oxidant sinks between measurements and models can be decreased by including forest floor emissions in these models.

The total VOC load in the atmosphere remains largely unknown because VOCs are difficult to measure and the boreal biosphere emits at least 25 different VOCs (Schallhart et al., 2018). VOCs are released by tree shoots (Aalto et al., 2014), stems (Vanhatalo et al., 2015) and forest floor (Aaltonen et al., 2013), and more specifically by decomposition processes, vegetative litter, root stores and plant VOC synthesis (Hayward et al., 2001; Leff and Fierer, 2008; Faubert et al., 2010). VOC synthesis of plants and microbes together with VOC volatilization are strongly affected by temperature (Guenther et al., 1993; Kesselmeier and Staudt, 1999). Temperature dependent VOC fluxes from the boreal forest floor (Aaltonen et al., 2013; Mäki et al., 2017) can increase when soil temperatures increase in a warming climate (Fan et al., 2014). Warming can also change vegetation cover and directly influence the abundance of the different VOC sources hence affect the amount and blend of emitted VOCs. In this study, we tested whether we could find evidence of such changes in an 8-year continuous data set of forest floor VOC fluxes. Previously reported measurements have been rather shortterm, and only few cover more than one growing season (Hellén et al., 2006; Asensio et al., 2007, 2008; Faubert et al., 2010).

Volatile organic compound fluxes between the forest floor and the atmosphere are dominated by isoprenoids (e.g., isoprene, monoterpenes, sesquiterpenes) and oxygenated VOCs (alcohols, aldehydes, and ketones) (Hellén et al., 2006; Aaltonen et al., 2011, 2013; Mäki et al., 2017). VOC fluxes from the boreal forest floor are highest during spring and autumn (Hellén et al., 2006; Aaltonen et al., 2011, 2013; Mäki et al., 2017). High spring fluxes are likely connected to snowmelt, which increases soil moisture and accelerates microbial decomposition and leaf growth by exposing soil and leaves to radiation. In the northern ecosystems, VOCs are produced by evergreen and deciduous dwarf shrubs (Rinnan et al., 2013), mosses (Hanson et al., 1999), roots (Hayward et al., 2001), and decomposing litter (Isidorov and Jdanova, 2002; Isidorov et al., 2016). A warmer climate will lead to an earlier snowmelt and growing season start, which can cause increased VOC flux in spring and possibly affect the emission blend. A warmer climate can also change precipitation patterns and affect VOC emissions, because soil water content impacts plant metabolism and microbial decomposition (Staudt et al., 2002; Davidson and Janssens, 2006). High monoterpene load from the soil surface into the atmosphere in autumn is likely released by decomposing and decaying pine litter (Mäki et al., 2017), which contains monoterpene storages (Kainulainen and Holopainen, 2002). VOCs can also be released by freeze–thaw and dry–wet cycles (Asensio et al., 2007, 2008; Insam and Seewald, 2010) in spring and autumn.

In this study, our aim was to use the long-term field measurements with automated chambers to determine the dynamics of forest floor VOC fluxes and to estimate the contribution of forest floor fluxes to the whole forest ecosystem fluxes. We analyzed the effects of environmental and site-specific variation (i.e., air and soil temperature, growing season length, soil moisture, and vegetation cover) on forest floor VOC exchange in order to develop a statistical model that could explain the interannual, seasonal, and daily patterns in emission rates.

## Materials and Methods

### Continuous Chamber Flux Measurements and Supporting Data

We measured the forest floor VOC fluxes in the southern boreal forest (established in 1962) at the SMEAR II (Station for Measuring Ecosystem-Atmosphere Relations) station (61°51′ N, 24°17′ E, 180 m above sea level). Canopy basal area is covered by *Pinus sylvestris* (75%), *Picea abies* (15%), and broadleaf species (10%) such as *Betula pendula* and *Sorbus aucuparia*. The forest floor is covered by ericoid shrubs (35.4%) such as *Vaccinium vitis-idaea*, *Vaccinium myrtillus*, and *Calluna vulgaris*, mosses (67.8%) such as *Pleurozium schreberi*, *Dicranum polysetum*, *Dicranum scoparium*, and *Hylocomium splendens*, tree seedlings (0.2%) such as *Sorbus aucuparia*, *Pinus sylvestris*, *Betula pendula*, and *Picea abies*, and grasses (8.4%) such as *Deschampsia flexuosa* and *Melampyrum sylvaticum* (Mäki et al., 2017). The soil type is Haplic podzol, and organic soil contains the largest proportion of carbon (356 mg g^-1^) and nitrogen (13 mg g^-1^) compared with mineral soil (5–32 and ∼1 mg g^-1^, respectively).

**FIGURE 1:**
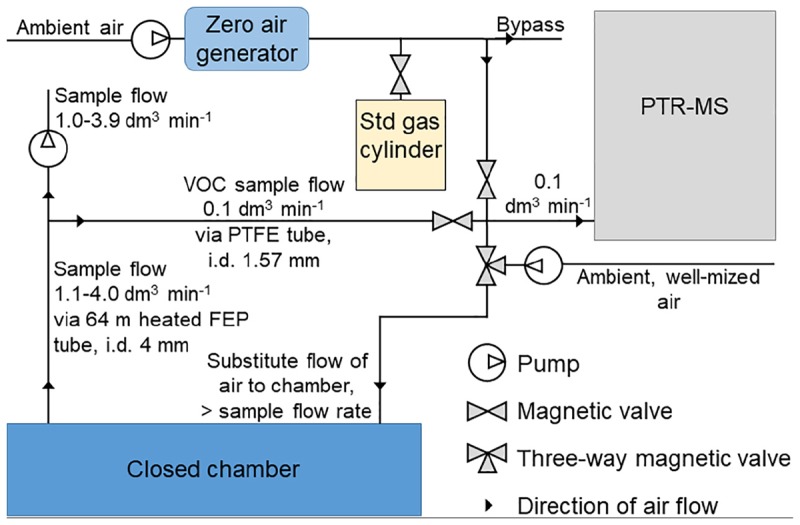
The measurement set-up of continuous chamber flux measurements.

We measured the VOC fluxes using three automated dynamic (flow-through) chambers (80 cm × 40 cm × 25 cm) installed on permanent stainless-steel soil collars (80 cm × 40 cm × 10 cm) every spring (Aaltonen et al., 2013). The shared volume of chamber and soil collar was 112 L. The automated chambers were connected to a quadrupole proton transfer reaction mass spectrometer (quadrupole PTR-MS, Ionicon Analytik, Innsbruck, Austria, de Gouw and Warneke, 2007) and VOC fluxes were measured from April 20, 2010 to November 7, 2017. Chamber frames were made of aluminum and the sides and top of the chamber were covered by a transparent fluorinated ethylene propylene (FEP) film (0.05 mm) (Aaltonen et al., 2013). Mixing the air with the chamber closed was done with two fans (wind speed 2.2 m s^-1^ and air volume of flow-through 167 L min^-1^) and the chamber opening direction was toward the south. We drew air samples from the chambers using a flow of 1.1–4.0 L min^-1^ through a 64 m heated FEP tube (inner diameter 4 mm) and determined the VOC concentrations from a side flow (0.1 L min^-1^) through a polytetrafluoroethylene (PTFE) tube (Aaltonen et al., 2013) (Figure 1). Chamber closure time was 15 min. To avoid underpressure inside the chambers, we substituted the sample flow with well-mixed ambient air using a 30–100% higher flow rate compared with the sampling flow. The VOC concentrations of the substitute air were measured immediately before every chamber enclosure. We measured the masses (amu) of methanol (33), acetaldehyde (45), acetone (59), isoprene (69), benzene (79), monoterpene fragment (81), methyl butenol (87), toluene (93), hexenal (99), hexanal (101), monoterpenes (137), and methyl salicylate (153). We measured all the masses every year, except benzene, methyl salicylate, hexenal, and hexanal between 2010 and 2012 and methyl butenol and toluene between 2013 and 2017. The calibration gas (Apel-Riemer Environmental, Inc., Denver, CO, United States) included methanol, acetaldehyde, acetone, isoprene, benzene, toluene, hexanal (except in summer 2010), and α-pinene. The chamber system was automated and every chamber was measured eight times a day (Aaltonen et al., 2013). The instrument was calibrated 1–4 times per month based on the scheme presented by Taipale et al. (2008). The quadrupole PTR-MS has a high sensitivity and a short response time (Taipale et al., 2008), suitable to quantify oxidized VOCs (Aaltonen et al., 2013), but is unable to separate compounds with the same molecular mass such as different monoterpenes.

We measured the forest floor carbon dioxide (CO_2_) and H_2_O fluxes from the same chambers using infrared light absorption analyzers (URAS4, Hartman and Braun, Frankfurt, Germany), until spring 2013, when the instrument was replaced with a LICOR LI-840A (LI-COR, Lincoln, NE, United States). We estimated the H_2_O and CO_2_ fluxes using the same mass balance equation (Kolari et al., 2012) that was used for the VOC flux calculations. Precipitation was monitored with an FD12P weather sensor (Vaisala Oyj, Helsinki, Finland) at a height of 18.0 m. Soil volumetric water content and soil temperature were measured from four to five pits and at each pit on every soil horizon (H horizon, eluvial E horizon, illuvial B horizon, and parent material C horizon). The mean thickness of the organic soil is 6.0 cm, the eluvial E horizon 2.0 cm and the illuvial B horizon 16 cm at the SMEAR II stand (Mäki et al., 2017). The soil moisture and soil temperature sensors were placed in the middle of each soil horizon. Soil temperatures were measured at 15-min intervals using silicon temperature sensors (Philips KTY81–110, Philips Semiconductors, Eindhoven, Netherlands). Volumetric water contents were recorded at 60-min intervals using the time-domain reflectometry method (TDR-100, Campbell Scientific, Ltd., United Kingdom). Photosynthetically active radiation (PAR) was measured using an LI-190 quantum sensor (Li-Cor Biosciences, Lincoln, NE, United States) above the canopy. The monthly total litterfall and the fraction of needles was determined with 21 L collectors (Mäki et al., 2017). We determined plant coverage (%) of ericoid shrubs such as *Vaccinium vitis-idaea* and *Vaccinium myrtillus*, mosses such as *Pleurozium schreberi*, *Dicranum polysetum*, *Dicranum scoparium*, and *Hylocomium splendens*, and other plant species such as *Linnaea borealis* and *Deschampsia flexuosa* for each measurement chamber based on visual assessment in 2010 and 2017 (Table 1). Total plant coverage of soil collars was 55–94% in 2017.

**Table 1:** Total plant coverage (%) and coverage (%) of *Vaccinium vitis-idaea*, *Vaccinium myrtillus*, *Linnaea borealis*, and mosses of three measurement chambers in 2010 and 2017.

	Chamber 1		Chamber 2		Chamber 3	
			
Year	2010	2017	2010	2017	2010	2017
Total plant coverage (%)	9^∗^	90	66^∗^	94	53^∗^	55
Coverage of *Vaccinium vitis-idaea* (%)	41^∗^	2	71^∗^	35	42^∗^	28
Coverage of *Vaccinium myrtillus* (%)	21^∗^	15	29^∗^	25	58^∗^	32
Coverage of *Linnaea borealis* (%)	38^∗^	28				
Mosses (%)		55		40		40


*Plant coverage was determined visually from photographs. ^∗^Aaltonen et al. (2013).*

### Continuous Ecosystem Flux Measurements

In order to assess the importance of forest floor fluxes to the total VOC budget of the stand, we compared the measured VOC fluxes from the forest floor with the total ecosystem fluxes. The data set of ecosystem fluxes was published in Rantala et al. (2014, 2015). Ecosystem fluxes of 27 different VOCs were measured with the profile method using the quadrupole PTR-MS (Ionicon Analytik GmbH, Innsbruck, Austria) with a 2-s sampling time from two heights in the canopy (4.2 and 8.4 m) and four heights above the canopy (16.8, 33.6, 50.4, and 67.2 m) (Rantala et al., 2014, 2015). Measurements were performed eight times a day using continuous flow (33 L min^-1^) through a 100 m long sample tube (PTFE, inner diameter 8 mm). The air sample entered the PTR-MS as a side flow through the 4 m long sampling tube (PTFE, inner diameter 1.6 mm) (Rantala et al., 2014). The quadrupole PTR-MS completed a whole measurement cycle within 1 h, in which the quadrupole PTR-MS ran through each measurement height nine times, with one individual repetition at each height taking 1 min (Rantala et al., 2014). Losses of monoterpenes and oxygenated VOCs in the tube walls are typically small (Kolari et al., 2012), because flow rate is relatively high and the ratio of the tube wall area and tube diameter is relatively small. We measured VOC fluxes for the whole ecosystem from May 28, 2010 to September 8, 2014. The calculations of 30-min average volume mixing ratios were described by Taipale et al. (2008). The VOC fluxes were estimated from the profile measurements using the surface layer profile method based on the Monin-Obukhov theory, and calculations were described in detail by Rantala et al. (2014). For the flux calculations, the ambient temperature was measured from each height using Pt-100 sensors, and turbulence parameters were determined using the three-dimensional acoustic anemometer (Solent 1012R2, Gill Instruments, Ltd., United Kingdom) at a height of 23 m.

### Chamber Flux Calculations

Flux calculation was based on the mass balance equation (Kolari et al., 2012). The rate of change of concentrations during the first 400 s as well as the known concentrations of substitute air were taken into account. We quantified the VOC fluxes according to the VOC concentration change (*C*) during the chamber closure, which is derived from the mass balance equation (Hari et al., 1999), Eq. 1:

(1) $$V\frac{dc}{dt}=E+F\left(C_{i}-C\right)$$

where *V* is the chamber volume, *E* is the VOC emission rate (positive flux) or the VOC uptake rate (negative flux), *F* is the flow rate of ingoing air, and *C_i_* is the VOC concentration of the air used to replace the sampled air volume. Calculating Eq. 1 for VOC concentration *C* as a function of time t after chamber closure leads to solution (Kolari et al., 2012):

(2) $$C(t)=C_{0}+(\frac{C_{i}-C_{0}}{V}+\frac{E}{F})\left(1-e^{-\frac{Ft}{V}}\right)$$

where *C*_0_ is the VOC concentration (μg m^-3^) at the time when the chamber was closed. The emission rate *E* (μg m^-2^ h^-1^) corresponds to the rate of change in concentration during the first 400 s after the chamber was closed (Eq. 2).

There was a negative exponential correlation between relative humidity (%) in the chamber headspace and water soluble VOC fluxes, and for this reason we only used methanol, acetone, and acetaldehyde fluxes measured under 75% relative humidity. This threshold led to removal of 74–81% of the methanol, acetone, and acetaldehyde fluxes for the chambers. All the data analyses for methanol, acetone, and acetaldehyde were performed using filtered data. Relative humidity inside the chamber headspace was measured right before the closure.

We tested the normality of different VOC fluxes measured from the automated chambers (*n* = 3) with the Kolmogorov–Smirnov test (df = 2). As the data were non-normally distributed, the statistical differences between years (Table 2) and between chambers (Supplementary Table S1) were determined using the non-parametric Kruskal–Wallis test (df = 2) at a significance level of < 0.05 (Supplementary Table S1).

**Table 2:** Start and end dates of volatile organic compound (VOC) flux measurements and total annual litterfall (g_DW_ m^-2^); environmental conditions calculated from March to October: annual mean temperature (°C), temperature sum (sum of daily mean temperatures > 5°C), mean soil moisture in the O horizon (m^3^ m^-3^), and precipitation sum (mm); timing of snowmelt and first snow in each year; annual mean monoterpene, methanol, acetone, and acetaldehyde fluxes (μg m^-2^ h^-1^) with standard deviation of three measurement chambers between 2010 and 2017 at the SMEAR II station.

	2010	2011	2012	2013	2014	2015	2016	2017
Start date	20.4	4.5	26.4	16.4	25.3	13.4	18.8	8.5
End date	12.11	2.12	12.6	8.10	19.8	20.11	28.11	26.10
Total litterfall	120	183	213	204	221	190	118	190
Temperature mean	9.0	10.1	10.5	8.7	9.7	9.0	8.9	7.3
Temperature sum (May–August)	1234	1202	981	1217	1122	1004	1111	883
Temperature sum (Mar–Oct)	1404	1469	1169	1433	1359	1236	1298	1030
Soil moisture	0.25	0.21	0.36	0.28	0.27	0.28	0.18	0.25
Precipitation sum	368	412	497	308	353	309	410	414
Snowmelt date	14.4	20.4	29.4	20.4	23.3	16.4	15.4	2.5
Snowfall date	15.10	28.11	26.10	18.10	18.10	19.11	3.11	26.10
**VOCs**								
Monoterpenes	17.0 (14.5)	9.2 (4.7)	7.2 (3.4)	16.6 (6.9)	8.6 (1.8)	11.8 (7.1)	19.1 (7.0)	8.3 (3.3)
Methanol	2.7 (5.3)	3.1 (9.3)	2.4 (6.0)	-0.9 (7.0)	1.4 (7.6)	1.1 (7.7)	-0.8 (5.4)	3.3 (4.0)
Acetone	0.9 (2.0)	1.6 (3.2)	0.8 (0.2)	-0.1 (2.8)	1.4 (2.9)	0.5 (2.0)	-0.2 (0.8)	0.7 (2.7)
Acetaldehyde	1.9 (2.1)	1.6 (1.8)	1.7 (0.6)	0.9 (0.2)	1.2 (0.8)	1.3 (1.5)	0.7 (0.1)	1.0 (1.4)


*Snowmelt date is when snow cover is mainly melt.*

**FIGURE 2:**
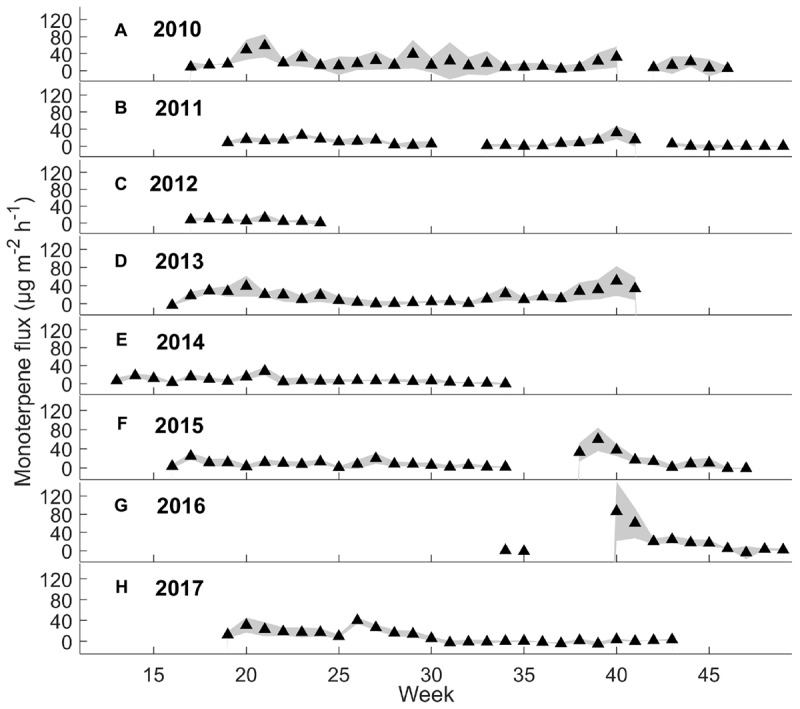
Weekly mean monoterpene fluxes (μg m^-2^ h^-1^) and standard deviation (*n* = 3 chambers) from 2010 to 2017.

**FIGURE 3:**
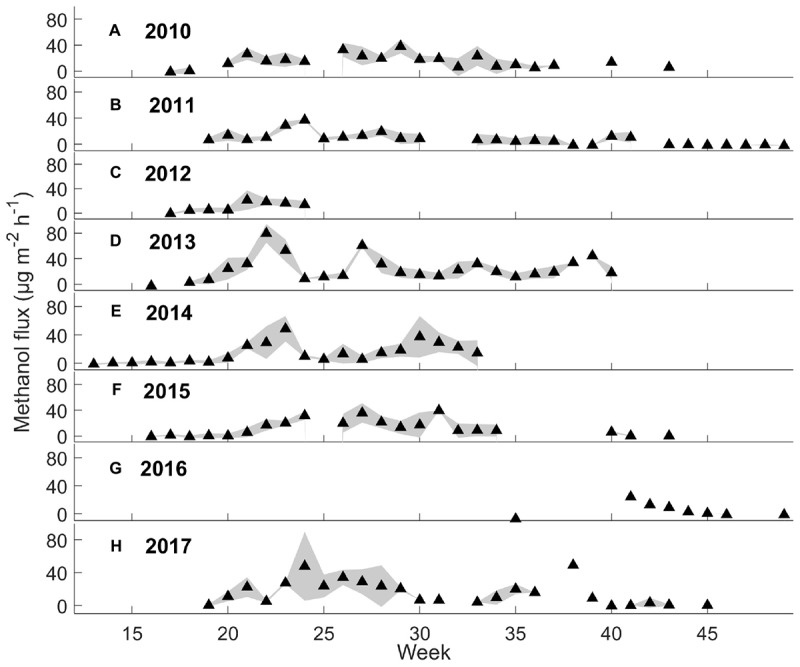
Weekly mean methanol fluxes (μg m^-2^ h^-1^) and standard deviation (*n* = 3 chambers) during daytime (9 a.m. to 8 p.m.) from 2010 to 2017 by filtering out the deposition first (see section “Chamber Flux Calculations”).

**FIGURE 4:**
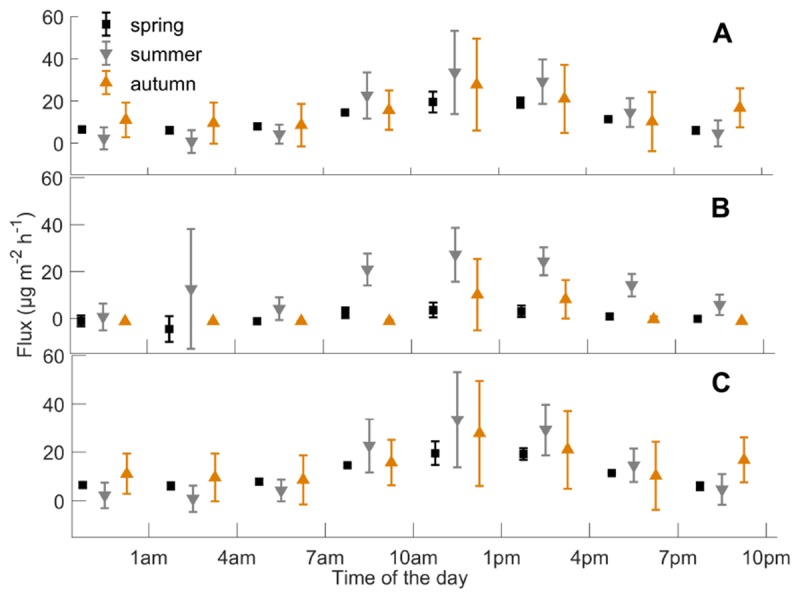
Mean **(A)** monoterpene, **(B)** methanol, and **(C)** acetone fluxes (*n* = 3 chambers), and standard deviation (μg m^-2^ h^-1^) during different times of the day from the boreal forest floor in spring, summer, and autumn, from 2010 to 2017. Summer starts when daily mean temperature is over 10°C, and autumn begins when daily mean temperature is below 10°C.

**FIGURE 5:**
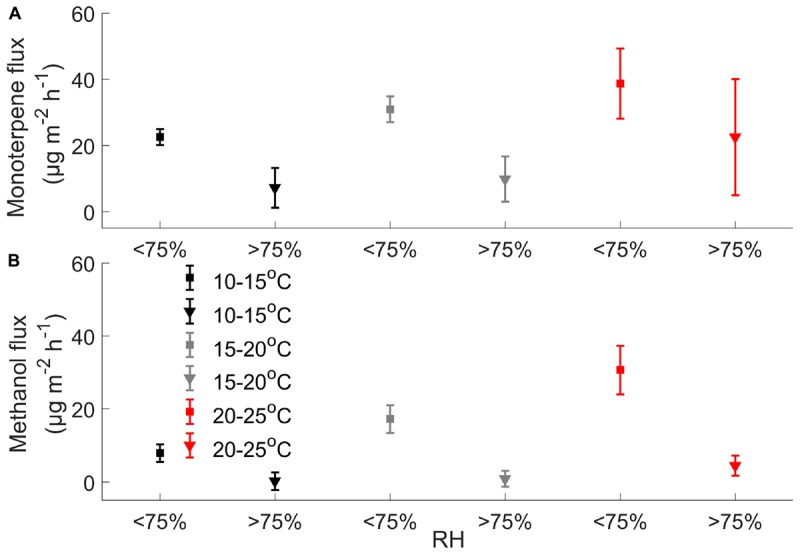
Effect of relative humidity on mean **(A)** monoterpene and **(B)** methanol fluxes (μg m^-2^ h^-1^) when chamber temperature is 10–15, 15–20, and 20–25°C (*n* = 3 chambers) using unfiltered data.

Weekly mean monoterpene fluxes were estimated using the whole data set (Figure 2) and weekly mean methanol fluxes using daytime measurements from 9 a.m. to 8 p.m. (*n* = 3) (Figure 3), because nighttime measurements were mainly filtered away with a 75% relative humidity threshold. We determined weekly mean methanol fluxes using nighttime measurements from 9 p.m. to 8 a.m. (Supplementary Figure S1). The mean monoterpene, methanol, and acetone fluxes were estimated at different times of day from the boreal forest floor in spring, summer, and autumn (Figure 4) based on the assumption that summer starts when daily mean temperature is over 10°C, and autumn begins when daily mean temperature is below 10°C. We determined the effect of chamber temperature and relative humidity on VOC fluxes from the forest floor (Figure 5 and Supplementary Figure S2).

**FIGURE 6:**
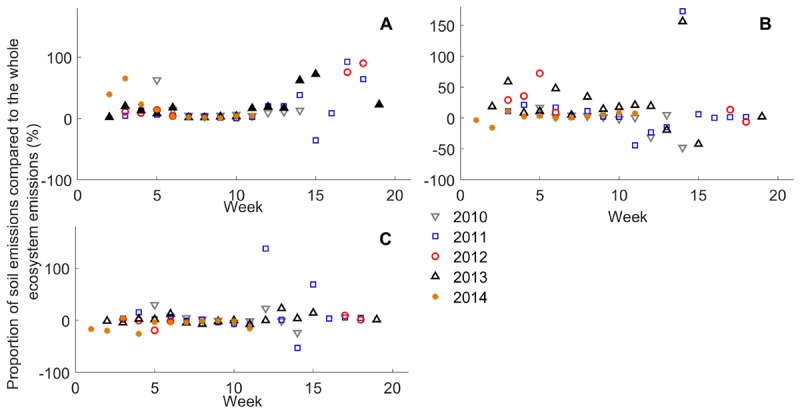
Proportion of 2-week cumulative sum of **(A)** monoterpene, **(B)** methanol, and **(C)** acetone fluxes (*n* = 3) from the forest floor compared with the 2-week cumulative sum of forest stand fluxes (%) from 2010 to 2014. Fluxes were compared using the whole data set without filtering with 75% relative humidity.

The cumulative sum (μg m^-2^ h^-1^) of methanol (33 amu), acetaldehyde (45 amu), acetone (59 amu), and monoterpenes (137 amu) for each 2-week period was calculated for each chamber and for the whole ecosystem using an unfiltered data set. The mean of the cumulative sums of three chambers was compared with the total ecosystem fluxes by calculating the proportion of soil fluxes relative to the whole ecosystem fluxes for each 2-week period (Figure 6).

### The Mixed Effects Linear Model

We developed a mixed effects linear model to determine which parameters can be used to explain the fluxes of the different VOCs. Model parameters are presented in Supplementary Table S2. We used the effect of chamber temperature, relative humidity, and above-canopy PAR to model monoterpene, methanol, acetone, and acetaldehyde fluxes. VOC fluxes (*V*) were modeled by the mixed effects linear model (*3*):

(3) $$V=B_{0}+B_{t}+B_{R}+B_{tR}+\in ,$$

where *B*_0_ represents a fixed intercept parameter, *B*_t_ represents fixed unknown parameters associated with chamber temperature, *B*_R_ represents fixed unknown parameters associated with relative humidity of the chamber, and *B*_t_*R* represents fixed parameters for interaction of the measurement chamber with relative humidity and temperature. In the model (*3*), the error term ∈ is presumed to have the form:

(4) $$\in =\propto_{CR}+\propto_{CT}+u,$$

where ∝ *CR* represents random parameters related to interaction of the measurement chamber (1, 2, and 3) and relative humidity of the chamber, ∝ *CT* represents random parameters related to interaction of the measurement chamber and chamber temperature, and *u* is an unobservable random error term.

### Accuracy of the Chamber Measurement Method

We tested the chamber wall effects in laboratory conditions by flushing gas containing the study compounds into the chamber headspace, and the concentrations of ingoing and outgoing air were determined by sampling air into the Tenax TA-Carboback-B adsorbent tubes. Concentration of the calibration gas in the ingoing air was from 1.7 to 3.1 μg m^-3^, varying with the compounds. The chamber was placed on a flat surface covered with a transparent FEP film (0.05 mm); air (flow rate 3 dm^3^ min^-1^) was moving into and out of the chamber through FEP tubes (inner diameter 6 mm), and we drew air samples from a side flow using a flow of 0.1–0.15 dm^3^ min^-1^. We determined the concentrations of individual monoterpenes (α-pinene, camphene, β-pinene, Δ3-carene, *p*-cymene, 1,8-cineol, limonene, terpinolene, linalool, and myrcene) and sesquiterpenes (longicyclene, isolongifolene, β-caryophyllene, aromadendrene, and α-humulene) from the adsorbent tubes by using a thermodesorption instrument (PerkinElmer TurboMatrix 650, Waltham, MA, United States) coupled with a gas chromatograph (PerkinElmer Clarus 600, Waltham, MA, United States) with a mass selective detector (PerkinElmer Clarus 600T, Waltham, MA, United States) (Mäki et al., 2017). Relative standard deviation (RSD, %) was calculated as a standard deviation between the four parallel samples taken during the chamber enclosure in the laboratory using constant concentrations of VOCs of ingoing air. RSD shows that the error of sampling and analytical method (thermal desorption–gas chromatography–mass spectrometry) was relatively low for both monoterpenes (5–14%) and sesquiterpenes (6–9%) (Supplementary Table S3).

## Results

### Interannual, Seasonal, and Diurnal Dynamics in Soil VOC Exchange

#### Interannual Dynamics

Forest floor VOC exchange was dominated by monoterpenes and oxygenated VOCs such as methanol, acetone, and acetaldehyde, while isoprene fluxes were mainly close to zero. Annual mean monoterpene fluxes ranged from 7 to 19 μg m^-2^ h^-1^, methanol fluxes from -1 to 3 μg m^-2^ h^-1^, acetone fluxes from 0 to 2 μg m^-2^ h^-1^, and acetaldehyde fluxes from 1 to 2 μg m^-2^ h^-1^ (Table 2). The VOC fluxes showed comparable emission rates between years.

#### Seasonal Dynamics

Interestingly, the seasonal dynamics of monoterpene fluxes from the forest floor differed from those of oxygenated VOCs. The highest weekly mean monoterpene fluxes were observed from the forest floor in spring (May–June) and in autumn (September–October) (maximum 59 and 86 μg m^-2^ h^-1^, respectively) (Figure 2). Weekly mean methanol fluxes were highest in spring and summer (May–August) (Figure 3: maximum 24 and 79 μg m^-2^ h^-1^, respectively) based on daytime measurements from 9 a.m. to 8 p.m. We found that acetone and acetaldehyde fluxes followed a similar seasonal pattern to that of methanol. Acetone and acetaldehyde fluxes were clearly lower than monoterpene fluxes but were at the same level as methanol fluxes (Table 2).

#### Diurnal Dynamics

We determined the diurnal dynamics of the forest floor VOC fluxes in spring, summer, and autumn. The diurnal maximum fluxes for monoterpenes, methanol, and acetone were observed in spring (19, 4, and 3 μg m^-2^ h^-1^), summer (33, 27, and 9 μg m^-2^ h^-1^), and autumn (28, 10, and 1 μg m^-2^ h^-1^), respectively (Figure 4). The monoterpene and methanol fluxes were strongest during the daytime in all seasons, while acetone and acetaldehyde fluxes showed clear diurnal dynamics only in spring and summer. Relatively high monoterpene, methanol, and acetone fluxes were also observed from 6 p.m. to 8 p.m. in summer.

### Temperature and Relative Humidity Effect on Forest Floor VOC Exchange

Our results showed that monoterpene, methanol, and acetone fluxes correlate with chamber temperature (Supplementary Figure S2). Chamber temperature explained 14–61% of methanol and 25–57% of acetone fluxes in spring and in summer (Supplementary Figure S2), but not in autumn, except in chamber 1 (55 and 51%). Monoterpene emissions showed correlation (7–50%) with chamber temperature from spring to summer and weak correlation in autumn (2–10%) (Supplementary Figure S2). Relative humidity had a significant effect on monoterpene and methanol fluxes (Figure 5).

### Mixed Effects Linear VOC Emission Model

**FIGURE 7:**
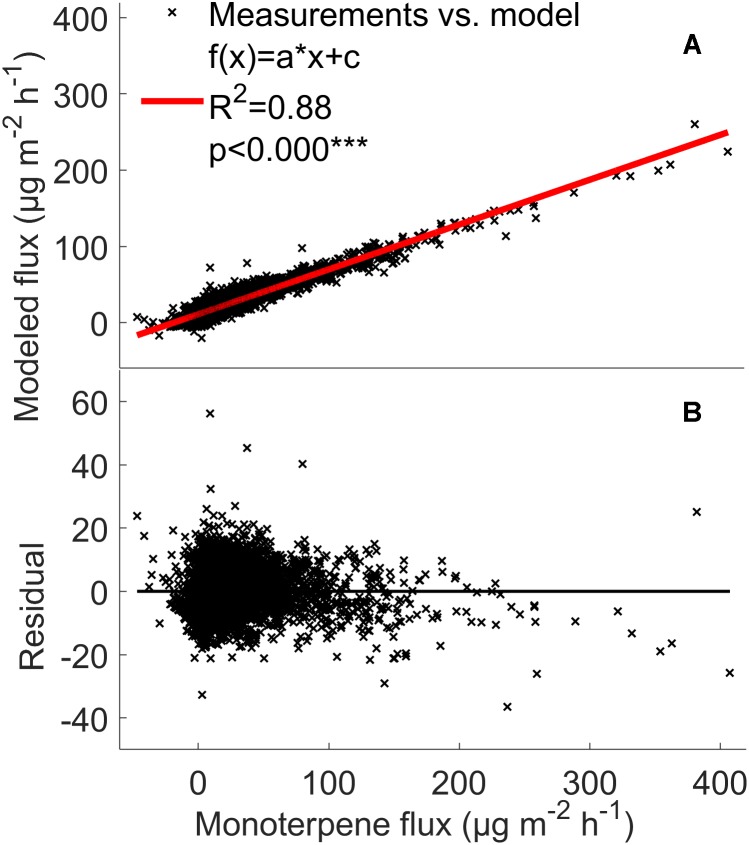
Comparison between the measured monoterpene **(A,B)** fluxes (μg m^-2^ h^-1^) from the soil chambers and the fluxes calculated using the mixed effects linear model with linear fit and residuals. Red line: measured flux = modeled flux.

**FIGURE 8:**
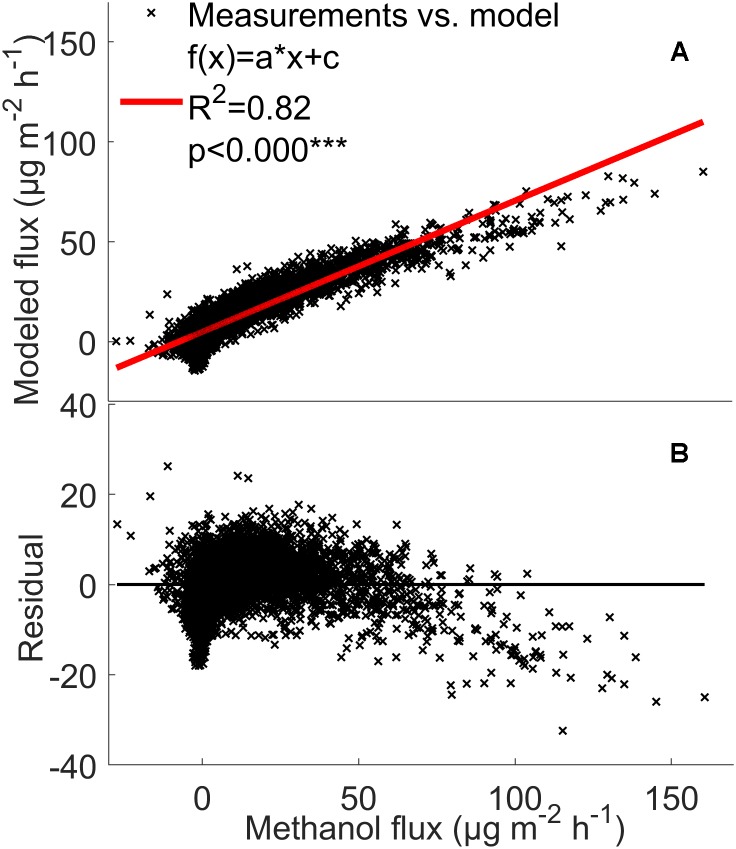
Comparison between the measured methanol **(A,B)** fluxes (μg m^-2^ h^-1^) from the soil chambers and the fluxes calculated using the mixed effects linear model with linear fit and residuals. The model was calculated by filtering out the deposition first (see section “The Mixed Effects Linear Model”) from all three chambers between 2010 and 2017. Red line: measured flux = modeled flux.

The model analysis showed that the VOC flux rates from the forest floor are indeed strongly affected by temperature and relative humidity. The model explained 79–88% of monoterpene, methanol, acetone, and acetaldehyde fluxes from the boreal forest floor (Figure 7, 8 and Supplementary Figure S3). This fairly simple statistical modeling approach is able to capture the individual behavior of each measurement chamber.

### Importance of Forest Floor to Ecosystem VOC Exchange

We compared forest floor VOC emissions to the whole ecosystem emissions measured by the flux gradient method between 2010 and 2014 (Rantala et al., 2014, 2015). The forest floor accounted for 2–93% of monoterpene fluxes relative to forest stand fluxes in spring and autumn and 1–72% of methanol fluxes in spring and early summer (Figure 6). The role of forest floor in forest stand fluxes was only a few percent in summer. Fluxes were compared using the whole data set without filtering with 75% relative humidity. VOC deposition dominated in the forest floor or forest stand during some 2-week periods (negative values in Figure 6).

### Uncertainties in VOC Exchange Measurements From the Forest Floor

The chambers differed from each other in emission rates but showed very similar and systematic temporal dynamics. Monoterpene and methanol fluxes were highest from chamber 1, where temperature was the highest (Supplementary Table S1). The acetaldehyde and acetone fluxes were mainly highest from chamber 1, but the differences between chambers were small. Chamber temperature was strongly correlated with ambient air.

## Discussion

### Interannual Dynamics

Our 8-year long data series of forest floor VOC exchange showed relatively small variation between the years. Annual mean monoterpene fluxes were very similar to earlier studies performed on the boreal forest floor (Aaltonen et al., 2011: α-pinene 0–14 μg m^-2^ h^-1^; Mäki et al., 2017: total monoterpenes 23 μg m^-2^ h^-1^; Wang et al., 2018: total monoterpenes 3–10 μg m^-2^ h^-1^). Also, mean fluxes of oxygenated VOCs, methanol, acetone, and acetaldehyde, were similar to earlier measurements at the same site in 2010 (Aaltonen et al., 2013: methanol -0.6 to 7.2 μg m^-2^ h^-1^; acetone -0.8 to 2.2 μg m^-2^ h^-1^; acetaldehyde 0.8–2.2 μg m^-2^ h^-1^).

Most of the interannual variability in monoterpene exchange resulted from variations in the temperature sum and precipitation between the years, while a similar trend was not observed for methanol, acetone, and acetaldehyde. Periods of high precipitation decreased monoterpene fluxes, which were seen also in low annual mean fluxes in 2011, 2012, and 2017. The effect of precipitation was assumed to be due to increased relative humidity and formation of water films on soil and leaf surfaces, which decreases VOC evaporation from surfaces. Annual mean monoterpene flux was also high in 2016 due to high monoterpene fluxes in October, which were likely released by fresh decomposing litter (Mäki et al., 2017; Wang et al., 2018).

### Seasonal Dynamics

The strong seasonal variation on monoterpene and VOC fluxes most probably results from seasonality in the production of VOCs via plant VOC synthesis (Aaltonen et al., 2011; Faubert et al., 2012) and litter decomposition (Hayward et al., 2001; Greenberg et al., 2012; Mäki et al., 2017). Temperature regulates plant VOC synthesis (Guenther et al., 1993), and hence this could explain the higher annual mean monoterpene fluxes during the warm summers (2010 and 2013) compared to the cold summers (2012 and 2017) (Table 2).

At our site, Vanhatalo et al. (2015) observed bursts of monoterpene from Scots pine stems during spring recovery in April. Similarly, a release of monoterpenes from belowground storages such as roots (Hayward et al., 2001) could explain the high forest floor monoterpene emissions during spring. Based on our study, it remains unclear whether monoterpenes were mostly emitted by litter or by roots, while the production in plants was likely small due to the fact the monoterpene emissions from all three chambers were similar in magnitude despite the differences in plant species coverage. Vegetation in chamber 1 was dominated by mosses that emit mostly isoprene and oxygenated VOCs and only minimal amounts of monoterpenes (Hanson et al., 1999; Hellén et al., 2006), while vegetation in chambers 2 and 3 were mostly dominated by *Vaccinium* spp. that emits monoterpenes (Aaltonen et al., 2011; Faubert et al., 2012). There were hardly any VOC flux measurements directly after snowmelt, while Hellén et al. (2006) found that seasonally highest monoterpene fluxes from the boreal forest floor were measured right after snowmelt at our measurement site. High monoterpene emissions in autumn are linked to decomposing litter (Greenberg et al., 2012; Mäki et al., 2017) and the organic soil, which contains easily available carbon for microbial decomposition.

Seasonality of oVOC fluxes in the forest floor was comparable to those of the forest stand fluxes. Gross primary production of ground vegetation is generally highest from mid-June to mid-August at our site (Kolari et al., 2006). The maximum forest floor methanol fluxes coincide with the maximum biomass production, which suggests that new surface vegetation growth is a major methanol source at our measurement site. The seasonality of forest floor methanol emissions follows the seasonality of canopy methanol exchange (Aalto et al., 2014) indicating that the processes and sources of methanol are the same both in the canopy and in the forest floor. Other evidence is that oxygenated VOC fluxes were lowest from chamber 3 with the lowest vegetation cover from June to August (Supplementary Table S1). Methanol can also be released by microbes that synthesize VOCs (Bäck et al., 2010; Mancuso et al., 2015)especially in summer, when decomposer activity is high due to temperature driven enzyme activity (Davidson and Janssens, 2006). Methanol fluxes in early autumn are probably related to plant senescence – leaf litter is a strong methanol source (Warneke et al., 1999) and the senescence of *Vaccinium myrtillus* leaves is in early autumn (Kulmala et al., 2008).

### Diurnal Dynamics

The diurnal dynamics in forest floor VOC exchange were clearly affected by temperature and radiation changes between the daytime and nighttime, which corresponds with the earlier observations at the site (Aaltonen et al., 2013). Monoterpene synthesis in plants is typically light and temperature dependent (Kesselmeier and Staudt, 1999), and similar light and temperature dependent VOC production has been demonstrated with ericoid shrubs, grasses, and mosses (Hellén et al., 2006; Faubert et al., 2010; Aaltonen et al., 2013; Lindwall et al., 2015; Kramshøj et al., 2016). Monoterpene and methanol fluxes are affected by temperature due to the volatility of these compounds (Guenther et al., 1993) and by radiation that heats leaf surfaces. Methanol fluxes are also strongly dependent on stomatal conductance (Nemecek-Marshall et al., 1995), which is high during daytime when plants maintain their photosynthesis by taking up CO_2_ through stomata and maintain water transport through transpiration.

Nighttime monoterpene emissions observed in spring and autumn (Figure 4) were likely affected by soil processes independent from light availability. Oxygenated VOC fluxes were close to zero in the nighttime, but we also observed deposition on leaf surfaces and chamber walls. Night-time deposition and daytime emissions of methanol were also observed from the bare temperate cropland in spring (Bachya et al., 2018). In our study, the nighttime deposition of methanol, acetone, and acetaldehyde is linked to lower temperatures and higher humidity in the chamber headspace. VOCs were likely released from the aqueous layer on plant and soil surfaces in the morning, when air humidity drops.

### Modeling Soil VOC Exchange

Chamber temperature explained monoterpene, methanol, and acetone fluxes from the forest floor (Supplementary Figure S2), similar to methanol and acetone fluxes from *Pinus sylvestris* shoots, which correlated with temperature (Aalto et al., 2014). Temperature dependence of monoterpenes is not as clear, because monoterpenes are emitted immediately from synthesis or with a delay from storage structures in resin ducts, glandular trichomes, or other storage structures (Laothawornkitkul et al., 2009). Litter also contains monoterpene storage structures and accelerates microbial decomposition (Hayward et al., 2001; Isidorov and Jdanova, 2002; Leff and Fierer, 2008; Gray et al., 2010; Isidorov et al., 2016). Monoterpene release from litter is likely directly linked to temperature due to missing monoterpene synthesis. However, low correlation between chamber temperature and monoterpene fluxes gives an indication that monoterpenes are released from and consumed simultaneously in the forest floor. Ramirez et al. (2010) showed that soil can be a sink for VOCs released by litter, like methanol that is used as a carbon source by bacteria via the ribulose monophosphate cycle and the serine cycle (Quayle and Ferenci, 1978). Different abiotic and biotic stresses also affect plant VOC emissions (Baldwin et al., 2006; Loreto and Schnitzler, 2010; Niinemets et al., 2013), which can make temperature responses of forest floor fluxes more complex.

The mixed effects linear model accounting for location dependent variability, temperature, and relative humidity was able to explain 79–88% of monoterpene, methanol, acetone, and acetaldehyde fluxes from the boreal forest floor (Figure 7, 8 and Supplementary Figure S3). It seems that with such a multi-annual data set, a fairly simple statistical modeling approach is able to capture individual behavior of each measurement chamber, while the challenge remains how to capture the spatial variation of soil VOC exchange. This model is not directly suitable to estimate forest floor VOC exchange in other ecosystems based on ambient temperature and relative humidity. The model clearly underestimates high methanol, acetone, and acetaldehyde fluxes, indicating that environmental factors other than temperature and relative humidity are the main drivers of these fluxes in the chambers. One likely reason is deposition of these water soluble molecules on moist leaf and chamber surfaces. Deposition should be modeled with a different mixed effects linear model, because the processes behind VOC deposition are different than the biological and physicochemical processes, which regulate production and evaporation of VOCs.

Volatile organic compound fluxes seemed to be strongly stimulated by temperature when the relative humidity effect is included (Figure 7, 8). VOC fluxes were observed to decrease with increasing relative humidity (Figure 5). Therefore our results give an indication that VOC fluxes from the boreal forest floor will likely increase in a warming climate. The global mean surface temperature is expected to increase by a minimum of 0.3–1.7°C under Representative Concentration Pathway (RCP) 2.6 and a maximum of 2.6–4.8°C under RCP8.59 by 2100 (IPCC Fifth Assessment Report (AR5), 2014). A temperature increase of 2°C can lead to a twofold increase in monoterpene fluxes and a five-fold increase in sesquiterpene fluxes in subarctic ecosystems (Valolahti et al., 2015). In our study, monoterpene fluxes correlate with temperature in spring and summer, but the flux rate rise was smaller with increasing temperature, possibly because vegetation is less sensitive to temperature fluctuations in boreal ecosystems compared with arctic ecosystems.

In boreal ecosystems, a warming climate is expected to increase ericoid shrub cover and decrease moss cover, which can affect leaf litter quality and decomposition rates (De Long et al., 2016). Decomposition activity is expected to increase due to temperature dependent enzyme activity of microbes, and this can affect belowground VOC production. Warming can also affect plant VOC synthesis, release of evaporated VOCs through stomata, and the abundance of different plant species. The boreal forest floor is prevailingly covered by monoterpene-emitting *Vaccinium* spp. (Faubert et al., 2012; Aaltonen et al., 2013) at our site. *Vaccinium myrtillus* has shown a 36% increase in aboveground biomass by warming soil (Anadon-Rosell et al., 2014), which could make understory vegetation less diverse (Dawes et al., 2011) and impact monoterpene production in a warming climate.

### Forest Floor Affects Ecosystem Fluxes

The comparison indicated that the forest floor is a significant contributor to the whole ecosystem fluxes, but its importance varies between seasons, being most important in autumn. The forest floor covered only a few percent of the forest stand fluxes in summer. Our results are in line with those of Aaltonen et al. (2013), who showed that the forest floor covered from several percent to 10s of percent of the total ecosystem fluxes depending on the compound and the season. Our method of comparing the observed forest floor level VOC emissions with ecosystem level VOC fluxes does not include oxidation of VOCs taking place during the transport from the forest floor to the above-canopy atmosphere. Therefore our results presented in Figure 7 should be considered as order of magnitude estimates rather than definitive values determining the proportion of forest floor oriented VOCs.

The seasonal dynamics of oxygenated VOCs (methanol, acetaldehyde, and acetone) were similar between the forest floor and the whole ecosystem (data not shown), indicating that vegetation is likely a significant source of oxygenated VOCs from the forest floor. In trees, methanol production is connected to growth and is produced mainly in leaves and needles in spring, and in stem and root expansion in summer. Soil VOC production can also influence the canopy fluxes, because methanol produced in the stem and roots during their growth, can be transported to the canopy via transpiration stream (Rissanen et al., 2018), and hence be emitted through stomata (Folkers et al., 2008). Peaking acetone and acetaldehyde fluxes above a hardwood forest in autumn were speculated to result from leaf senescence and decaying biomass (Karl et al., 2003). Acetone and acetaldehyde are also produced in the air from the oxidation of other VOCs.

Our results show that monoterpene fluxes from the forest floor contributed the highest load (9–93%) to the whole ecosystem fluxes in autumn due to low canopy fluxes. Monoterpenes are likely released from decomposing litter and plant senescence, because VOC synthesis of ground vegetation likely decreased similarly to shoot VOC emissions. This can provide a significant contribution to the OH sink in boreal forest air. VOCs affect formation and oxidation processes of OH radicals and ozone (Jacob et al., 2005; Hüve et al., 2007). Monoterpenes, isoprene, and other organic compounds were calculated to cover about 24% of the total OH reactivity in August based on a one-dimensional vertical chemistry-transport model (Mogensen et al., 2011). Later, it was estimated that monoterpenes cover ∼14% and oxidized VOCs ∼44% of OH radical sinks (Mogensen et al., 2015). Ozone reacts mainly with inorganic compounds and only ∼3% with monoterpenes and 6% with sesquiterpenes and is deposited on the soil and vegetation surfaces (Mogensen et al., 2015; Zhou et al., 2017).

### Uncertainties

Temporal dynamics of forest floor VOC exchange can rather well be captured with three chambers, while several factors may affect and increase the uncertainties in chamber measurements. Our results indicate that vegetation cover affects BVOC emission rates and together with varying temperature creates spatial variation in the fluxes. The contribution of light to soil VOC fluxes is relatively small due to poor light availability under a closed canopy. Monoterpenes can also stick on lipophilic plant leaves (Mäki et al., 2017), which could explain why the highest fluxes were observed from chamber 1 with the lowest *Vaccinium* spp. coverage.

The quadrupole-PTR-MS is an instrument with high sensitivity and short response time and it has been shown to be able to perform online-measurements of reactive trace gasses from grasslands (Pape et al., 2009). Flux measurements of water soluble VOCs such as methanol, acetone, and acetaldehyde are sensitive to biases, because they tend to be adsorbed on moist surfaces (vegetation, soil, and chamber walls). For this reason, soil chamber data of water soluble VOCs was filtered with 75% relative humidity. A relative humidity of 70% in the chamber has been recommended by Kolari et al. (2012). Relative humidity of the headspace is often over 75% when measurements are performed during the nighttime or rainy days, meaning that water soluble VOCs are dissolved in moist surfaces on chamber walls and leaves. Seasonal fluxes of oxygenated VOCs were slightly overestimated, because the flux measurements performed during the nighttime or rainy days are underrepresented in the data. There were also gaps in the data due to technical analyzer problems, which affected the data coverage between years. Data were missing from mid-June to October in 2012, from September to October in 2014, and from April to mid-August in 2016.

We found hardly any correlation between the VOC fluxes and soil temperature. This may be caused by the spatial variation in soil temperature, and the lack of soil temperature sensors installed right next to the soil chambers, and in the top-most litter layer, where most of the soil-emitted VOCs are released (Hayward et al., 2001; Greenberg et al., 2012). Moreover, the effect of soil temperature on VOC fluxes may be hidden by the complexity of different VOC sources and their different responses to temperature.

## Conclusion

We used the 8-year data set to assess the interannual, seasonal, and diurnal dynamics of forest floor VOC fluxes and compared them with the simultaneously measured total ecosystem fluxes. The forest floor affects ecosystem VOC exchange, emitting 2–93% of monoterpene fluxes in spring and autumn and 1–72% of methanol fluxes in spring and early summer. Oxygenated VOC fluxes showed similar seasonal dynamics between the forest stand and the forest floor.

The highest weekly mean monoterpene fluxes were observed from the forest floor in spring and autumn (maximum 59 and 86 μg m^-2^ h^-1^, respectively) and the highest weekly mean methanol fluxes in spring and summer (maximum 24 and 79 μg m^-2^ h^-1^, respectively). The seasonal dynamics indicate that litter and ground vegetation were the dominant sources of monoterpenes and oxygenated VOC from the boreal forest floor. Forest floor VOC exchange was dominated by monoterpenes and methanol and the flux dynamics was relatively similar between the 8 years, whereas the emission rates differed between the chamber locations. Accounting for location dependent variability and temperature and relative humidity, a mixed effects linear model was able to explain 79–88% of monoterpene, methanol, acetone, and acetaldehyde fluxes from the boreal forest floor. Forest floor VOC exchange was measured in the prevailing climate, but based on temperature responses of monoterpenes, and especially oxygenated VOCs, it seems that forest floor VOC fluxes will likely increase in warming climate.

Forest floor VOC exchange should be measured using continuous long-term measurements in the different ecosystems to define the contribution of soils to ecosystem VOC exchange and hence their effect on atmospheric processes globally.

## Data Availability

The data of forest floor VOC fluxes for this study can be found in the Supplementary Material.

## Author Contributions

MM was responsible for preparing the manuscript. All authors participated in planning the experiment, analyzing data, preparing the manuscript, and reviewed the manuscript.

## Conflict of Interest Statement

The authors declare that the research was conducted in the absence of any commercial or financial relationships that could be construed as a potential conflict of interest.
