# Supplementary material for: Interannual and Seasonal Dynamics of Volatile Organic Compound Fluxes From the Boreal Forest Floor
Source: Front Plant Sci. 2019 Feb 22;10:191. doi: 10.3389/fpls.2019.00191 (PMC6395408; doi:10.3389/fpls.2019.00191)
Supplement: Supplementary file 4 [file Data_Sheet_4.pdf]

Supplementary Material

Interannual and seasonal dynamics of volatile organic compound fluxes from the boreal forest floor

***Mari Mäki, Juho Aalto, Heidi Hellén, Mari Pihlatie, and Jaana Bäck**

*** Correspondence:** mari.maki@helsinki.fi

1 Supplementary Data

The data of forest floor VOC fluxes is available online.

Data sheet number describes the measurement location (chamber number). Data_Sheet_1-3.CSV files include the following **columns**: **1**=Year, **2**=Month, **3**=Day, **4**=Hour, **5**=Minute, **6**=Second, **7**=Prevailing temperature in the chamber (°C), **8**= Prevailing relative humidity in the chamber (%), **9**= flux of the mass 33 (µg m^{-2} h^{-1}), **10**= flux of the mass 45 (µg m^{-2} h^{-1}), **11**= flux of the mass 59 (µg m^{-2} h^{-1}), **12**= flux of the mass 69 (µg m^{-2} h^{-1}), **13**= flux of the mass 79 (µg m^{-2} h^{-1}), **14**= flux of the mass 81 (µg m^{-2} h^{-1}), **15**= flux of the mass 99 (µg m^{-2} h^{-1}), **16**= flux of the mass 101 (µg m^{-2} h^{-1}), **17**= flux of the mass 137 (µg m^{-2} h^{-1}), and **18**= flux of the mass 153 (µg m^{-2} h^{-1}).

2 Supplementary Figures and Tables

Table S1. Chamber temperature (°C) and monthly mean monoterpene, acetone, methanol, and acetaldehyde fluxes (µg m^{-2} h^{-1}) from the three chambers between 2010 and 2017. Standard deviations are shown in brackets. The effect of measurement location (chamber) on fluxes was tested with the Kruskal–Wallis test. Significant differences between the chambers are indicated by different letters ($p<0.05$). There were no measurements in January and February and very few in November and December.

	**Chamber temperature**			**Methanol**		
**Month**	Chamber 1	Chamber 2	Chamber 3	Chamber 1	Chamber 2	Chamber 3
March	4.8 (2.6)	5.5 (1.9)	3.7 (2.1)	−0.2^{a} (2.3)	−1.1 (0.8)	−1.7^{b} (1.5)
April	7.3 (4.2)	7.0 (3.6)	7.1 (4.2)	2.3^{a} (3.8)	−0.9^{b} (3.1)	0.4^{c} (2.1)
May	13.4 (6.3)	13.3 (5.3)	12.7 (5.7)	17.7^{a} (22.5)	10.7^{b} (19.0)	13.4^{b} (22.3)
June	17.4^{a} (5.9)	16.7^{a} (4.8)	15.3^{b} (5.1)	24.3^{a} (25.8)	20.4 (22.0)	16.6^{b} (16.5)
July	22.3^{a} (5.0)	20.4^{b} (4.0)	20.1^{b} (4.0)	32.3^{a} (23.2)	21.6^{b} (18.3)	12.4^{c} (10.4)
August	20.0^{a} (4.0)	17.7^{b} (3.3)	18.3^{b} (3.7)	21.3.^{a} (12.7)	9.0^{b} (11.7)	6.5^{b} (6.8)
September	14.6^{a} (4.1)	9.7^{b} (4.8)	12.7^{c} (2.8)	18.4^{a} (12.4)	3.9^{b} (8.0)	4.2^{b} (6.0)
October	4.3^{a} (3.7)	3.3^{a} (2.7)	5.2^{b} (3.1)	4.9^{a} (8.9)	0.0^{b} (2.2)	0.5^{b} (2.8)

	**Monoterpenes**			**Acetaldehyde**		
**Month**	Chamber 1	Chamber 2	Chamber 3	Chamber 1	Chamber 2	Chamber 3
March	15.4^{a} (9.6)	5.2^{b} (5.9)	6.4^{b} (7.2)	0.9^{a} (1.2)	−0.2 (0.9)	−0.1^{b} (1.0)
April	13.1^{a} (15.9)	11.7^{a} (14.3)	15.1^{b} (13.6)	1.7^{a} (2.1)	0.9^{b} (2.1)	0.6^{b} (1.6)
May	22.5^{a} (40.4)	15.3^{b} (27.0)	13.6^{b} (22.5)	4.8^{a} (6.7)	4.4^{a} (4.8)	3.2^{b} (4.6)
June	19.3^{a} (39.4)	8.0^{b} (20.5)	9.7^{c} (17.9)	6.7^{a} (6.4)	5.0^{b} (4.7)	3.9^{c} (3.2)
July	18.2^{a} (35.8)	5.7^{b} (17.9)	7.6^{b} (19.8)	5.0^{a} (4.8)	5.4^{b} (3.8)	4.1^{a} (2.6)
August	10.8^{a} (23.4)	2.2^{b} (12.9)	2.2^{b} (10.6)	3.7^{a} (2.3)	3.6^{b} (4.1)	2.2^{c} (1.8)
September	27.9^{a} (42.5)	10.2^{b} (20.9)	11.1^{c} (20.0)	1.8^{a} (1.6)	0.9^{b} (1.4)	0.7^{b} (1.2)
October	25.4^{a} (31.9)	11.5^{b} (38.3)	7.9^{c} (20.8)	−0.3^{a} (0.6)	−0.1^{b} (0.4)	−0.3^{a} (0.5)
	**Acetone**					
**Month**	Chamber 1	Chamber 2	Chamber 3			
March	0.4^{a} (1.8)	−1.6^{b} (1.7)	−1.0^{b} (0.9)			
April	2.2^{a} (3.6)	−0.2^{b} (2.0)	0.5^{b} (2.7)			
May	5.9^{a} (9.2)	5.0^{a} (7.0)	3.4^{b} (5.4)			
June	6.1^{a} (9.1)	5.0^{a} (5.3)	2.9^{b} (4.1)			
July	6.4^{a} (8.6)	5.9^{a} (5.6)	4.4^{b} (5.1)			
August	3.0^{a} (4.3)	2.1^{b} (3.8)	1.6^{b} (2.7)			
September	1.7^{a} (1.8)	0.1^{b} (1.0)	−0.9^{c} (1.8)			
October	−0.1^{a} (1.9)	−0.8^{a} (0.7)	0.1^{b} (1.1)			

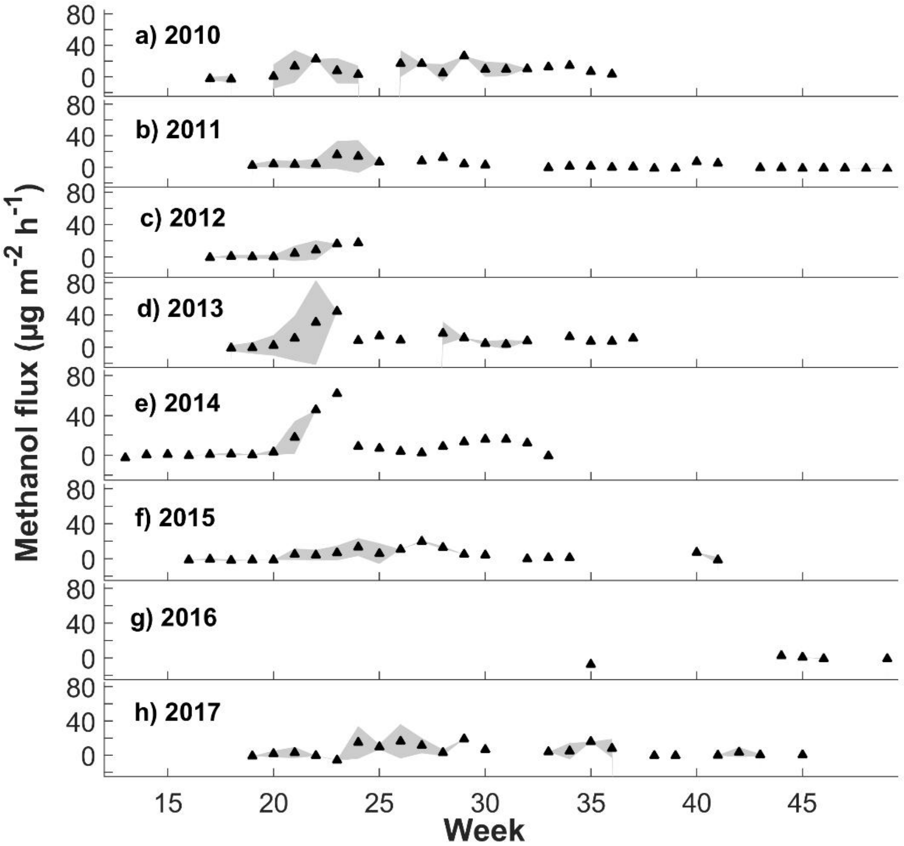

Figure S1. Weekly mean methanol fluxes (μg m^{-2} h^{-1}) and standard deviation (n = 3) during nighttime (9 p.m. to 8 a.m.) from 2010 to 2017 by filtering out the deposition first.

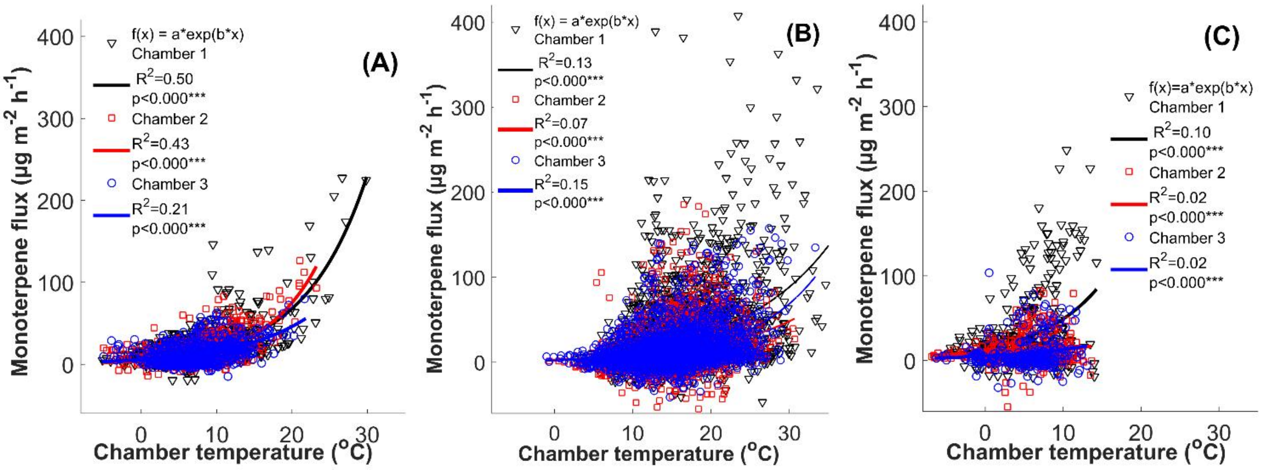

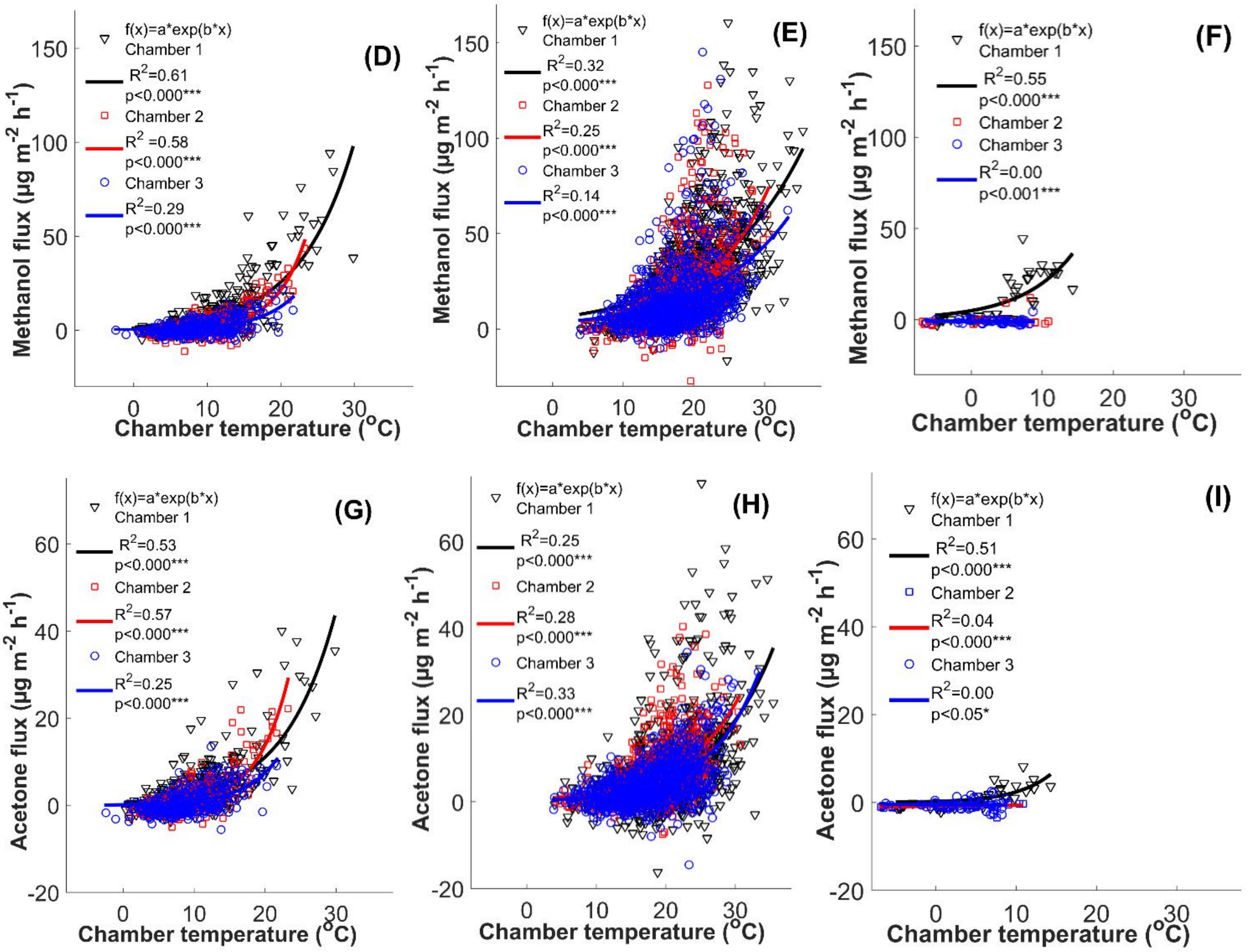

Figure S2. Relationship of monoterpene (A–C), methanol (D–F), and acetone (G–I) fluxes (µg m^{-2} h^{-1}) and chamber temperature (°C) in spring (A, D and G), summer (B, E and H), and autumn (C, F and I) between 2010 and 2017. Summer begins when daily mean temperature rises over 10°C, and autumn begins when daily mean temperature decreases under 10°C. Only daytime measurements (above-canopy PAR >50 µmol m^{-2} s^{-1}) were used and methanol and acetone fluxes were filtered with 75% relative humidity.

Table S2. Parameters of the mixed effects linear model of forest floor volatile organic compound (VOC) fluxes. CI = confidence interval; RH = relative humidity; T = temperature. The significance of the effect was described using p<0.1 (o), p<0.05 (*), p<0.01 (**), p<0.001 (***)).

Model: VOC ~ 1 + chamber T + chamber RH + (1 | chamber:chamber T) + (1 | chamber:chamber RH)

MONOTERPENES

	DF	AIC	BIC	LogLik	Deviance
	4814	46569	46608	-23278	46557

Fixed effects coefficients (95% CIs):

	Estimate	Lower	Upper	p
Chamber T	42.28	36.81	47.74	<0.001***
Chamber RH	1.41	1.28	1.53	<0.001***

Chamber T * chamber RH	-0.60	-0.68	-0.52	<0.001***	
	Random effects covariance parameters (95% CIs):				
	Estimate	Lower	Upper		
Factor (chamber) * chamber T	8.81	5.67	13.68		
Factor (chamber) * chamber RH	19.31	17.16	21.73		
Error	21.86	19.63	24.33		
	**ACETONE**				
	DF	AIC	BIC	LogLik	Deviance
	4835	29182	29221	-14585	29170
	Fixed effects coefficients (95% CIs):				
	Estimate	Lower	Upper	p	
Chamber T	8.64	7.75	9.52	<0.001***	
Chamber RH	0.39	0.37	0.41	<0.001***	
Chamber T * chamber RH	-0.17	-0.19	-0.16	<0.001***	
	Random effects covariance parameters (95% CIs):				
	Estimate	Lower	Upper		
Factor (chamber) * chamber T	1.48	0.92	2.36		
Factor (chamber) * chamber RH	2.85	2.09	3.90		
Error	3.76	3.12	4.52		
	**METHANOL**				
	DF	AIC	BIC	LogLik	Deviance
	4835	39708	39747	-19848	39696
	Fixed effects coefficients (95% CIs):				
	Estimate	Lower	Upper	p	
Chamber T	17.43	14.80	20.05	<0.001***	
Chamber RH	1.43	1.37	1.49	<0.001***	
Chamber T * chamber RH	-0.40	-0.43	-0.36	<0.001***	
	Random effects covariance parameters (95% CIs):				
	Estimate	Lower	Upper		
Factor (chamber) * chamber T	4.68	3.15	6.95		
Factor (chamber) * chamber RH	7.73	5.17	11.55		
Error	11.55	9.55	13.97		
	**ACETALDEHYDE**				
	DF	AIC	BIC	LogLik	Deviance
	4835	25716	25755	-12852	25704
	Fixed effects coefficients (95% CIs):				
	Estimate	Lower	Upper	p	
Chamber T	6.52	5.90	7.14	<0.001***	
Chamber RH	0.31	0.30	0.33	<0.001***	
Chamber T * chamber RH	-0.12	-0.13	-0.11	<0.001***	
	Random effects covariance parameters (95% CIs):				

	Estimate	Lower	Upper
Factor (chamber) * chamber T	1.21	0.85	1.72
Factor (chamber) * chamber RH	1.59	0.95	2.67
Error	2.81	2.37	3.35

Table S3. Testing the chamber wall effects in laboratory conditions: standard deviation between the four parallel samples of the chamber enclosure using constant volatile organic compound concentration of ingoing air (relative standard deviation (RSD), %) in February 2015. Samples were analyzed using thermal desorption–gas chromatography–mass spectrometry. RSD shows the error of sampling and analytical method.

**Monoterpenes**	**RSD (%)**
α-pinene	12
camphene	13
β-pinene	11
Δ3-carene	10
p-cymene	10
1,8-cineol	13
limonene	12
terpinolene	6
linalool	5
myrcene	14
**sesquiterpenes**	**RSD (%)**
longicyclene	7
isolongifolene	6
β-caryophyllene	7
aromadendrene	9
α-humulene	6

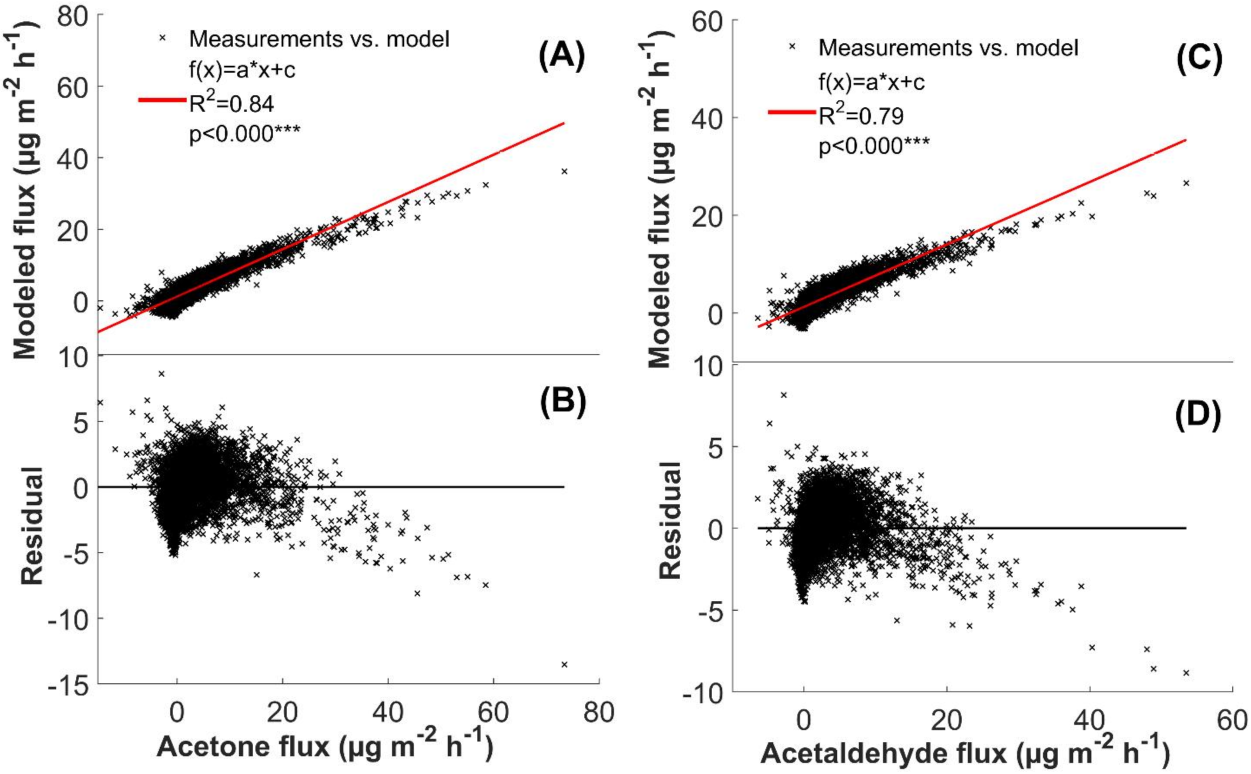

Figure S3. Comparison between the measured acetone (A–B) and acetaldehyde (C–D) fluxes (µg m^{-2} h^{-1}) from the soil chambers and the fluxes calculated using the mixed effects linear model with linear fit and residuals. The model was calculated by filtering out the deposition first from all three chambers between 2010 and 2017. Red line: measured flux = modeled flux
